# Intravenous Injection of Milk

**Published:** 1878-07-20

**Authors:** 


### INTRAVENOUS INJECTION OF MILK.

### A SUBSTITUTE FOR TRANSFUSION.

[New York Medical Journal, May 1878.]

Dr. T. G. Thomas, in a paper with the above title, puts forward very elegant reasons why hope should not be abandoned in the class of cases where transfusion of blood, although indicated, is impracticable, until the simple operative procedure of injecting milk into a vein has been tried. He gives first a short *resume* of the history of transfusion, and then shows that milk is not after all, so very different from blood, or from the chyle which is poured into the blood. He finds that Dr. Hodder, of Toronto, first resorted to the injection of milk in 1850, in three moribund cases of Asiatic cholera, and that two of them recovered. A year ago Dr. Howe, of New York, pursued the same course in a case of inanition accompanying tubucular disease, but without more than ephemeral success.

Without detailing Dr. T.'s cases, we will only say that he has employed the method in three cases. One made an excellent recovery, the second died of exhaustion, attending suppuration, and the third of prolonged interstitial hemorrhage. The life of the first was certainly saved, while a longer lease of life was given to the other two than they would have otherwise enjoyed.

Dr. T. alluded to the experiments of Dr. Howe, upon dogs, and accounted for his uniform failure on the ground that he had used milk which had stood for several hours; whereas it was most essential that the milk should be absolutely fresh. The author sums up these propositions:

1. injection of milk is feasible and safe.

2. Only milk removed from a healthy cow, and within a few minutes of its use, should be used. Decomposed milk is as dangerous as decomposed blood.

2. A glass furnished with a rubber tube attached, ending in a very small cannula, the whole scrupulously clean, is in every respect preferable to the more elaborate apparatus used in transfusion.

4. The whole proceeding is vastly more simple than transfusion, and offers positively no difficulties.

5. The injection of milk, like that of blood, is commonly followed by a chill, and rapid rise of temperature; these symptoms, however, quickly subside, and improvement follows.

6. The measure is indicated not merely in cases prostrated by hemorrhage, but in disorders which greatly depreciate the blood, like cholera, pernicious anæmia, typhoid fever, and as a substitute for diseased blood.

7. Not more than eight ounces of milk should be injected at any one operation; this, however, may be repeated as occasion requires.

Any accessible vein may be selected; perhaps the cephalic is preferable.—*Chicago Med. Ex.*

---

### WARNING AGAINST THE HYPODERMIC USE OF MORPHIA.

Dr. Levinstein, of Berlin, says, in his late work on the "Morphia Mania," (*morphiumsucht*), that it is generally caused by hypodermic injections of the drug, given by the medical attendant during illness, to relieve pain, and continued by the patients themselves after the actual need of it has disappeared, for the sake of the mental excitement it produces. The danger involved was not generally suspected during the first years of the use of the new means of administration, but the existence of the Maison de Sante, under the medical direction of the author, which is devoted to the cure of sufferers from this craving, shows how large its evil effects must already be. The sufferers feel quite well for a certain time, while using the narcotic, but before long, evident symptoms of disease appear. The patients become emaciated, the complexion ashy or dark red, the eyes lack lustre, and vision is often deranged; thirst, nausea, and loss of appetite, constipation, and the secretion of albumen by the kidneys, impotence, a form of delirium tremens different from that caused by alcoholic excesses, intermittent fever, and great derangement of the moral character, are among the morbid appearances produced.—*Medical and Surgical Reporter.*

